# Underestimation of pelvic organ prolapse in the supine straining position, based on magnetic resonance imaging findings

**DOI:** 10.1007/s00192-018-03862-0

**Published:** 2019-01-17

**Authors:** Anique T. M. Grob, Judith olde Heuvel, Jurgen J. Futterer, Diana Massop, Angelique L. Veenstra van Nieuwenhoven, Frank F. J. Simonis, Carl H. van der Vaart

**Affiliations:** 1 MultiModality Medical Imaging (M3i), Technical Medical Centre, University of Twente, Carre Building, Drienerlolaan 5, 7522 NB Enschede, The Netherlands; 2 Magnetic Detection and Imaging (MD&I), Technical Medical Centre, University of Twente, Enschede, The Netherlands; 3 Robotics and Mechatronics, Faculty of Electrical Engineering, Mathematics and Computer Science, Technical Medical Centre, University of Twente, Enschede, The Netherlands; 4 Department of Radiology and Nuclear Medicine, Radboud University Medical Center, Nijmegen, The Netherlands; 5 Department of Gynaecology, Medisch Spectrum Twente, Enschede, The Netherlands; 6 Department of Gynaecology, Ziekenhuis Groep Twente, Hengelo/Almelo, The Netherlands; 7 Department of Gynaecology, University Medical Center Utrecht, Utrecht, The Netherlands

**Keywords:** Upright MRI, Prolapse, Strain, Extent, Pubococcygeal line

## Abstract

**Objective:**

Pelvic organ prolapse (POP) is clinically diagnosed in the supine position, where the effect of gravity is simulated by having the patients put strain on their pelvic floor. The objective of this study was to determine the degree of POP underestimation in the supine position based on magnetic resonance imaging (MRI) findings.

**Methods:**

This prospective study was conducted with symptomatic POP grade ≥ 2 patients. Fifteen female patients were examined with an MRI system that allows supine and upright imaging. The differences between supine and upright in distances of the bladder neck, cervix, and pouch of Douglas from the pubococcygeal line (PCL) were estimated, together with changes in the genital hiatal area. Patients were scanned at rest and during straining. All distances were compared using the Wilcoxon ranking test.

**Results:**

All mean distances from the PCL increased from the supine–strain to the upright–rest and from the supine–strain to the upright–strain position. These distances were found in the supine and upright positions: the bladder descended 1.3 cm to 1.4 cm, the cervix 1.1 cm to 2.2 cm, and the pouch of Douglas 0.8 cm to 1.5 cm respectively (all *p* values <0.05). The hiatal area was larger in the upright–strain position (mean 42.0 cm^2^; SD ±14.8) than during the supine–strain position (mean 33.5 cm^2^; SD ±14.5), with a *p* value of 0.02.

**Conclusion:**

Upright MRI scanning of patients with POP grade ≥ 2 both at rest and during straining shows a significantly larger extent of the prolapse than that observed during supine straining.

## Introduction

Pelvic organ prolapse (POP) is a common condition, affecting 25–41% of middle-aged and elderly women [1, 2]. Accurate staging of POP is critical for treatment assignment, including decisions regarding the type and extent of surgical correction. The currently recognized diagnostic gold standard, the POP quantification (POP-Q) staging system, is a physical examination. According to its instructions, it is performed with the patient in the supine position [3]. This can result in under-staging of POP, because the effect of gravity is neglected, which is a recognized limitation of the POP-Q [4]. To simulate the effect of gravity, the patients are instructed to put strain on their pelvic floor by performing a Valsalva maneuver. However, the effect of Valsalva on the extent of the POP is dependent on the instructions by the physician and the knowledge and ability of the patients to relax (strain) their pelvic floor muscles [5].

In symptomatic POP, additional imaging may alter the diagnosis based upon physical examination [6–8]. A tilting low-field magnetic resonance imaging (MRI) system has the benefit of upright scanning next to standard supine imaging, which is crucial for this research. Next to that, MRI is a non-invasive imaging modality without ionizing radiation that can visualize the extent of the pelvic organs, to add information to both the diagnosis and management of POP [9].

The objective of this study was to determine the degree of POP underestimation in the supine position based on magnetic resonance imaging (MRI) findings.

## Materials and methods

### Study design and population

This prospective study was conducted with symptomatic POP patients from the gynecology department of the Medisch Spectrum Twente (MST) hospital in Enschede and Ziekenhuis Groep Twente (ZGT) hospital in Hengelo, the Netherlands. The study was approved by the Medical Ethical Committee Twente, registered as NL57965.044.13 and all women gave written informed consent. Patients on the waiting list for surgery in 2017 were approached by their gynecologist or a researcher to participate in this study. There was no selection based on the type of prolapse, e.g., descensus uteri, cystocele or rectocele.

To be eligible, the patients had to meet the inclusion criteria: a symptomatic prolapse, > 18 years of age, and a confirmed prolapse POP-Q ≥ grade 2 in the supine position. POP-Q grading was also performed in a standing position. During POP-Q measurements in a standing position, a patient was standing on a small chair. As in normal POP-Q measurements, the hymen was the reference point to which the other points were compared. The measurements were taken with the pelvic floor at rest. The gynecologist was sitting on the floor during these measurements.

Women were excluded if they had a medical history of prolapse surgery, did not pass the MRI safety-screening test, were currently pregnant, were unable to stand for 15 min without assistance, or were not allowed to perform maximum straining (based on cardiac or pulmonary disease). Additionally, the current coil circumference (115 cm) limits the inclusion, as women with a jeans size above 44(EU) or 14 (USA) could not be positioned inside the coil.

### MRI examination and image reconstruction and analysis

Magnetic resonance images were acquired using a 0.25-T scanner (G-Scan; Esaote, Genoa, Italy) in the supine and the upright positions. First, multi-slice 2D T2-weighted fast spin echo (FSE) scans were performed in two directions during rest: midsagittal and transverse (parallel to the pubococcygeal line [PCL]). The parameters of these scans were: echo time/repetition time (TE/TR): 25/3,480 ms, reconstructed resolution: 1.3 × 1.3 mm^2^, slice thickness: 5 mm, number of slices: 11, total scan time: ≈2 min. After that, 2D balanced steady-state free precession (bSSFP) images were acquired in midsagittal and transverse (parallel to the PCL) planes during rest and straining with the following parameters: TE/TR: 3.5/7 ms, reconstructed resolution: 1.5 × 1.5 mm^2^, slice thickness: 15 mm, field of view (FOV): 400 × 400 mm^2^, FA: 70°/90° (sagittal/transverse), acquisition matrix: 160 × 160/212 × 210 (sagittal/transverse), total scan time: ≈2 s.

Patients were first scanned in an upright position, followed by supine scanning to prevent fainting caused by orthostatic hypotension. The scans were obtained in consecutive order: FSE (transverse and sagittal) followed by the dynamic sequences. Patients were instructed on how to perform a proper Valsalva maneuver beforehand. A correct Valsalva is performed by inhaling deeply, holding your breath, exhaling against a closed airway, and bearing down as though straining to initiate a bowel movement. During the dynamic scan they performed a Valsalva maneuver twice for at least 5 s, under constant encouragement of the researcher. This was done twice, because previous studies concluded that repeating the Valsalva maneuver increases the induced maximal strain [5, 10]. The researcher checked the effectiveness of the maneuver real-time on the MR images.

All clinical parameters were determined offline using RadiANT DICOM viewer (Medixant, Poznan, Poland) and MATLAB 2014b (the MathWorks Inc, Natick, MA, USA). bSSFP data were analyzed in both scanning planes with FSE data only for anatomical reference. Representative sagittal and transverse images at rest and at maximal straining were selected based upon visual inspection. The observer who drew the anatomical lines is a radiologist with 9 years of experience in abdominal radiology blinded to all clinical parameters.

In 2016, a standardized protocol for prolapse evaluation with MRI was developed as a European standard. It concerned imaging sequences in addition to useful landmarks to assess the grade of prolapse based on supine MRI [11]. The PCL, running from the inferior border of the pubic symphysis to the last coccygeal joint, is used as a reference line for measuring the organ prolapse, as it represents the level of the levator ani muscle. The PCL proved to have the highest inter- and intra-observer reliability of MRI measurements in women with POP of the anterior and middle compartment compared with all proposed reference lines in the literature with an intercorrelation coefficient (ICC) between 0.70 and 0.99 [12–14].

**Fig. 1:**
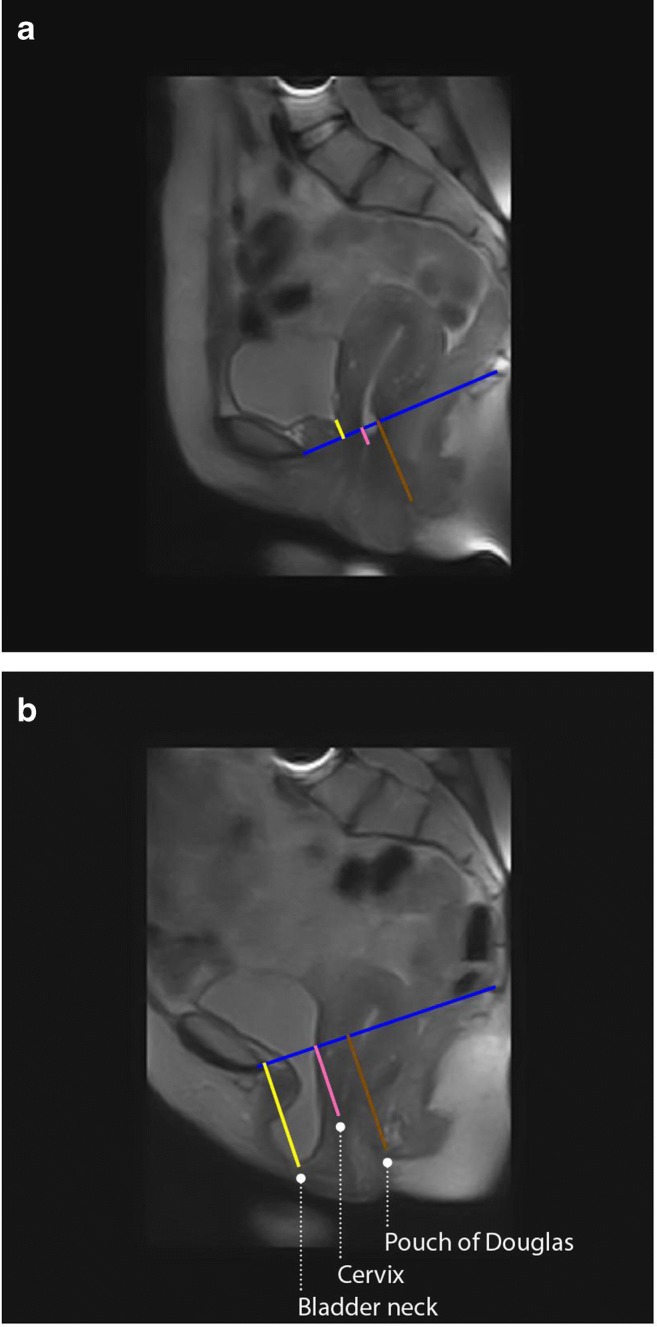
Sagittal MRI scan of a patient in **a** the supine–rest and **b** the upright–rest position. The *blue line* represents the pubococcygeal line (PCL). The *yellow*, *pink*, and *brown lines* visualize the distances of the PCL from the bladder neck, cervix, and pouch of Douglas respectively

From the PCL, the distances to the anterior part of the cervix, bladder neck, and pouch of Douglas were measured (Fig. 1). If these organs were located above the PCL, their distance had a negative value, according to international standards [15]. Therefore, all organs with a positive distance value indicated a prolapse. The hiatal area was calculated from a manually drawn contour on the transverse images at an internationally standardized level from the inner aspects of the pubococcygeus muscle [16].

### Statistical analysis

To determine the difference in prolapse extent between the supine and upright positions, the Wilcoxon signed rank test was performed. Statistical analysis was carried out using SPSS version 20.0 (SPSS, Chicago, IL, USA).

## Results

A total of 15 patients underwent MRI before pelvic floor surgery was performed. In one patient, the quality of the sagittal MR images was too low to analyze the images and in another patient the PCL–-cervix could not be measured because the patient had no uterus. No adverse events were reported.

Patient demographics are visualized in Table 1. Nine patients were diagnosed with POP grade 2, and 6 with grade 3, determined in the supine–strain position. In an upright position, 6 women stayed in grade 2 and 3 women stayed in grade 3. However, 3 women changed from grade 2 to 3 and 3 changed from grade 3 to 4. The planned surgery was either a Manchester–Fothergill or an anterior and/or posterior colporrhaphy.

**Table 1:** Patient demographics

	Mean (SD)
Age (years)	57.3 (8.6)
Body mass index (kg/m^2^)	26.1 (4.4)
Number of children	2.7 (0.8)
POP-Q grade supine	2.4 (0.5)
POP-Q grade upright	2.9 (0.7)

*POP-Q* pelvic organ prolapse quantfication system, *SD* standard deviation

**Fig. 2:**
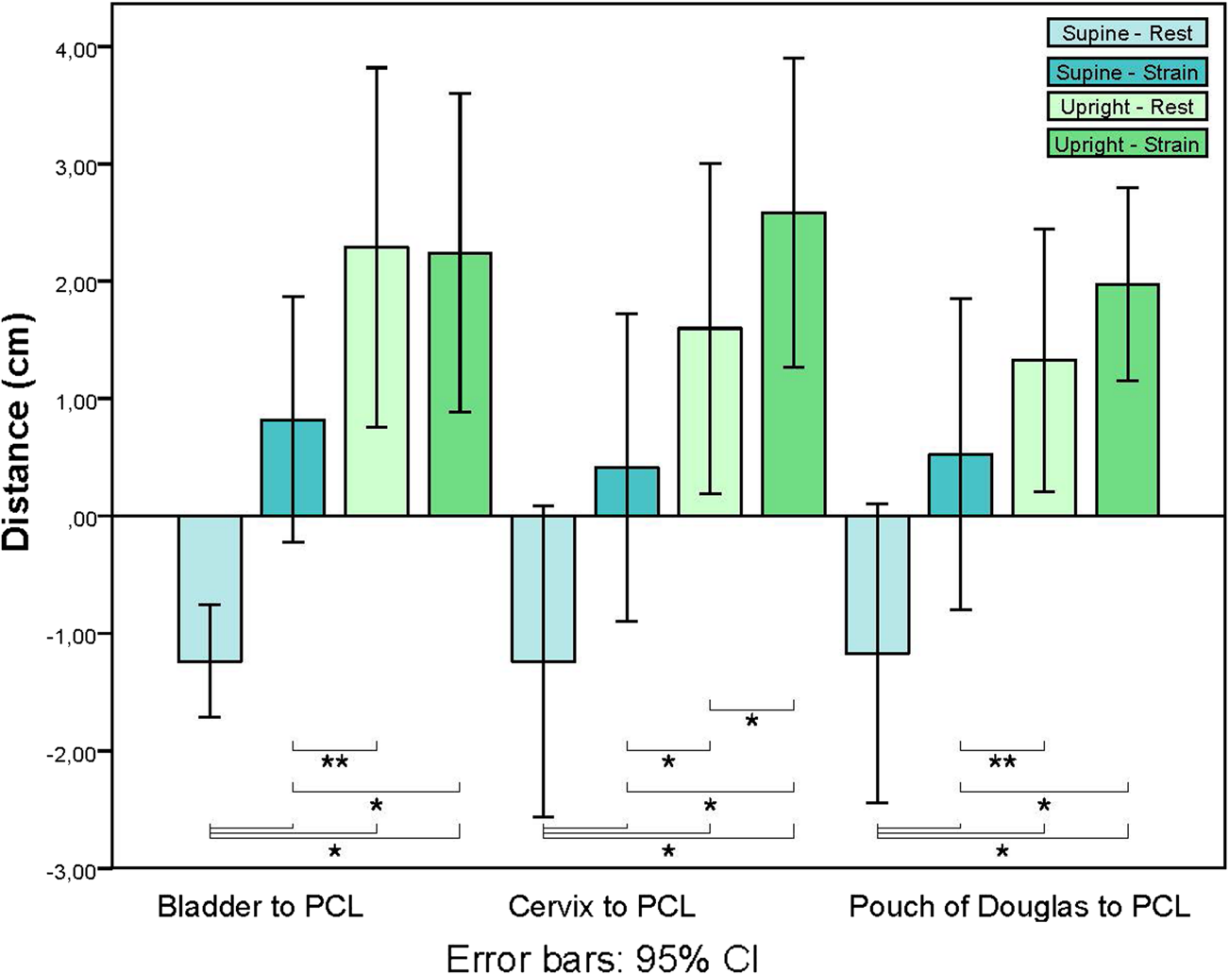
Boxplot of the distance between target organ and PCL. *CI* confidence interval. *p < 0.01; **p < 0.05

In Fig. 2, the mean distances of all patients from the bladder neck, cervix, and pouch of Douglas to the PCL in the supine and upright positions, during rest and strain, are shown with their 95% confidence interval (CI). All mean distances to the PCL increased from the supine–strain to the upright–rest and from the supine–strain to the upright–strain positions. These distances between different positions changed as follows: the bladder descended 1.3 cm and 1.4 cm respectively, the cervix 1.1 cm and 2.2 cm respectively, and the pouch of Douglas 0.8 cm and 1.5 cm respectively. The distance from all target organs to the PCL is significantly smaller in the supine–strain compared with the upright–rest and upright–strain positions (*p* < 0.05 or *p* < 0.01). The cervix to PCL distance was also significantly larger in the upright–strain compared with the upright–rest (*p* < 0.05) group. This difference was not found in the bladder neck to the PCL or pouch of Douglas to the PCL distances.

**Fig. 3:**
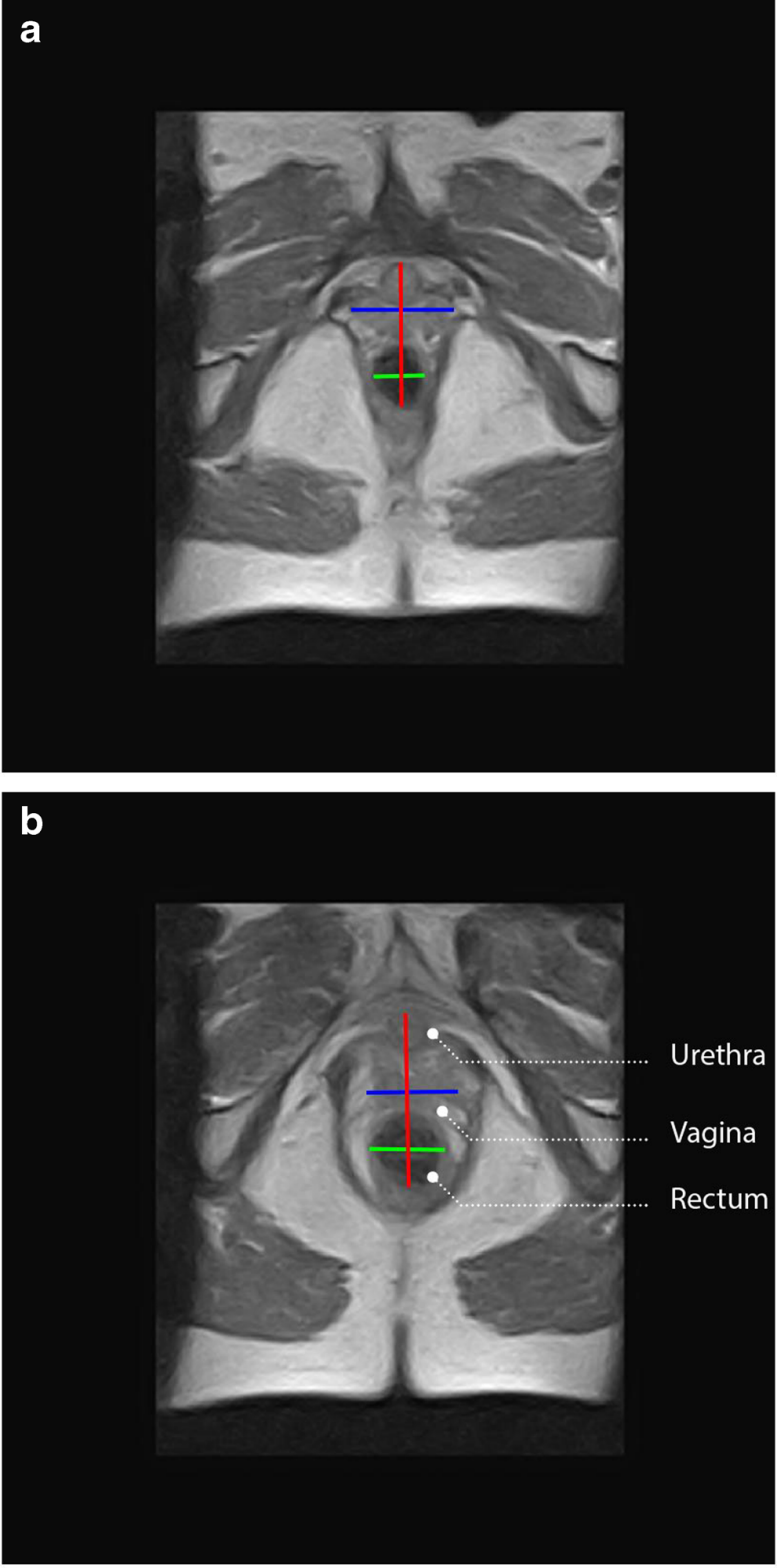
transverse MRI scan of a patient in **a** the supine–rest and **b** the upright–rest position. The *blue and green lines* represent the transverse diameter of the genital hiatus. The *red line* represents the anterior–posterior cross-section

**Fig. 4:**
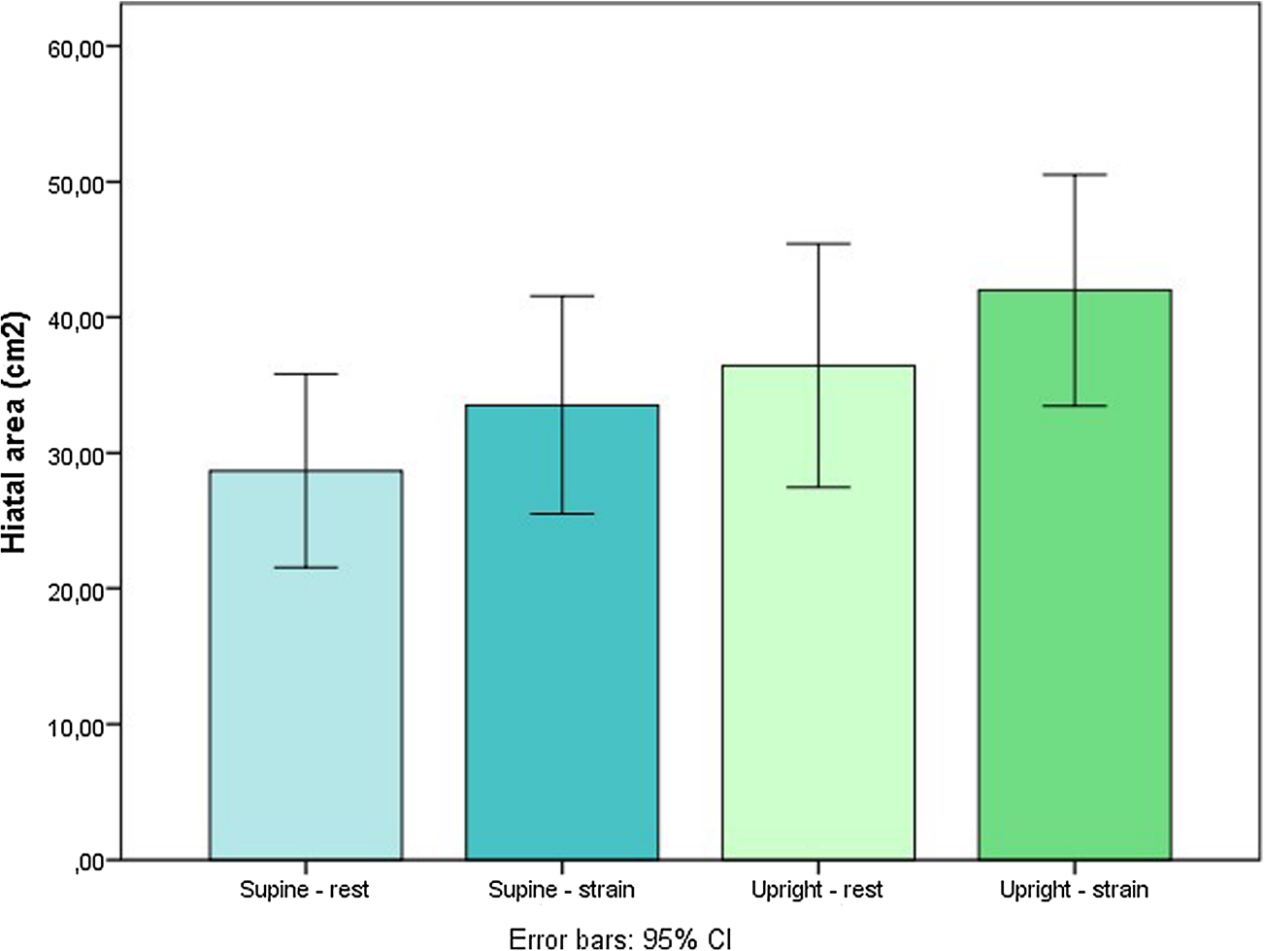
Boxplot of the difference in hiatal area in four different positions.*p < 0.01; **p < 0.05

Figure 3 shows an example of the change in hiatal area between the supine and the upright position. The Wilcoxon test showed no significant difference in hiatal area the between supine–strain and upright–rest positions. The hiatal area was significantly larger in the upright–strain (mean 42.0; SD 14.8) than in the supine–strain position (mean 33.5; SD 14.5), with a *p* value of 0.02 (Fig. 4).

## Discussion

In this study, MRI assessment revealed that the distance from the bladder neck, cervix, and pouch of Douglas to the PCL increased significantly in POP patients from the supine straining position relative to an upright rest and straining position. Additionally, in an upright straining position, the distance from the PCL to the cervix significantly increased compared with an upright–rest position. These findings indicate that patients diagnosed with POP ≥ grade 2 have a significantly larger extent of the prolapse measured on MR images in the standing position in comparison with the supine position. This increase in distance to the PCL was also found in the increase in POP-Q grade in 6 women in the standing position. The literature suggests that the supine position, which does not correspond to the situation in which the symptoms and discomfort of the prolapse is most severe, does not correlate with the true extent of the prolapse, confirmed by our findings [17, 18].

In accordance with the 2016 proposal for a standardized protocol of prolapse evaluation using MRI, we used the PCL as a reference to measure POP. One observer assessed the MRI data, as previous studies found that there is good to excellent interobserver agreement when evaluating POP on dynamic MRI for the PCL as a reference line, despite the difference in observer experience [15, 19]. Iacobellis and co-workers found that the positions of the bladder, vaginal fornix, and anorectal junction were significantly altered when the patient was imaged in the supine versus the sitting position. This occurred during rest and squeezing, and led to an underestimation of POP in the supine position [20]. Our study confirmed the findings of Iacobellis and co-workers. However, the study by Iacobellis examined women in a sitting position, which is not a maximum reflection of the contribution of gravity to the position of the pelvic organs. A recent study by Abdulaziz and co-workers also studied the maximal extent of prolapse, but focused on different reference lines. This study did not incorporate the effect of straining nor did it report the effect on different target organs of the reference lines [21].

Previous studies concluded that repeating the Valsalva maneuver increases the induced maximal strain [5, 10]. Interpreting the extent of the POP using this method will thus remain subjective. Our study results indicate that the prolapse extent during upright–rest examination is already larger than during supine straining. Determining prolapse in an upright–rest position could furthermore result in more reproducible results compared with straining by avoiding the effect that prolapse extent changes by the amount of straining maneuvers performed.

There are a few potential drawbacks of our method. First, the current image quality is not optimal because a receive-only coil was used, which is developed for imaging of the lumbar spine. Image quality may be further improved by developing a send and receive coil tailored to visualizing the pelvic area. Second, the current coil circumference limits the inclusion, as women with a jeans size above 44 (USA: size 14) needed to be excluded. As body mass index is known to be a risk factor for POP [22], being able to include women of all sizes would be of added value. Reliability of the image parameters could be a further improvement, by selecting the plane of minimal hiatal dimensions during straining. The dynamic MRI scans were planned at the level of minimal hiatal dimensions at rest, while the pelvic floor moves out of the plane during straining. This mismatch is mainly found on the transverse scans, but could be avoided if upright–rest scanning is considered for POP definition. Based on research outcomes, translation to clinical practice is indicated. However, the availability of the scanner in a hospital setting, its cost-effectiveness, and clinical pathways to include MRI in the POP decision process need to be further investigated. We hypothesize that MRI scanning might not be used as a routine examination in POP decision-making. Yet, we do see the possibility of implementing upright MR scanning in clinical practice in cases of complex, multi-compartment cases. MR scanning is already used in these cases; implementing upright scanning enables the most optimal image modality to be used. Finally, we did not study a possible relationship between the POP-Q grading and MRI findings. This was a deliberate consideration, given the reported disagreement between the two diagnostic methods [23, 24].

In this study, the clinical data of all 15 patients were generalized, i.e., all data, regardless of prolapse type and extent (descensus uteri, cystocele, rectocele or a combination), were analyzed as a single group. However, we were left with no other option owing to the limited number of patients with equally affected compartments and the same surgery type. If patients are selected according to their most affected compartment in subsequent research, the results might become even more striking. A larger cohort study is necessary to validate the findings of this case study.

This study shows the added benefit of using upright MRI in patients with POP. This modality also offers valuable research opportunities in the future. As no ionizing radiation is used, upright MRI can be deployed in longitudinal studies of prolapse progression and for monitoring the long-term effects of surgery. The additional information can be used to better diagnose the site and extent of POP and could even influence the choice of surgery type. Furthermore, other types of treatment, such as pessaries that currently suffer from fitting issues, may profit from 3D MR images of the pelvic floor in an upright position. This enables the opportunity for personalized pessary fitting.

In conclusion, upright scanning of patients with POP grade ≥ 2 at rest and during straining shows a significantly larger extent of the prolapse than that observed during supine straining. Upright MRI may therefore provide a greater insight into the true degree of prolapse in patients.
